# Limbic system associated membrane protein as a potential target for neuropsychiatric disorders

**DOI:** 10.3389/fphar.2013.00032

**Published:** 2013-03-26

**Authors:** Jürgen Innos, Kati Koido, Mari-Anne Philips, Eero Vasar

**Affiliations:** ^1^Department of Physiology, University of TartuTartu, Estonia; ^2^Estonian Academy of SciencesTallinn, Estonia

**Keywords:** limbic system-associated membrane protein, dopamine, 5-hydroxytryptamine, anxiety, genetic polymorphisms, major depressive disorder, panic disorder

## Abstract

The studies performed in laboratory animals and psychiatric patients suggest a possible role of limbic system-associated membrane protein (LAMP) in the mechanisms of psychiatric disorders. Stressful manipulations and genetic invalidation have revealed a role of the Lsamp gene in the regulation of anxiety in rodents. Besides that, Lsamp-deficient mice display reduced aggressiveness and impaired adaptation in novel and stressful environments. The behavioral effects of amphetamine were blunted in genetically modified mice. Recent pharmacological and biochemical studies point toward altered function of GABA-, 5-hydroxytryptamine-, and dopaminergic systems in Lsamp-deficient mice. Moreover, we found an association between the gene polymorphisms of LSAMP and major depressive disorder (MDD). Patients suffering from MDD had significantly increased ratio between risk and protective haplotypes of the LSAMP gene compared to healthy volunteers. However, the impact of these haplotypes for the function of LAMP is not clear and remains to be elucidated in future studies.

## INTRODUCTION

The limbic system-associated membrane protein (LSAMP) gene gives rise to LAMP, which is a 64- to 68-kDa heavily glycosylated protein, structurally characterized by three immunoglobulin (Ig) domains (Pimenta et al., 1996). LAMP protein is expressed on the surface of somata and proximal dendrites of neurons (Zacco et al., 1990) where it integrates via glycosyl-phosphatidyl-inositol (GPI) anchor (Pimenta et al., 1996). LAMP protein has been shown to be specific to the cortical and sub-cortical limbic-associated regions (e.g., perirhinal cortex, cingulate cortex, amygdala, hippocampus, and striatum) of the developing and adult brain (Levitt, 1984; Horton and Levitt, 1988; Pimenta et al., 1996; Reinoso et al., 1996). Despite the name, LAMP is not expressed only in the limbic-associated areas, but also less intensely in the midbrain and hindbrain regions (Reinoso et al., 1996). A 99% amino acid sequence identity between human and rodent LAMP (Pimenta et al., 1996) indicates strong phylogenetic conservation of the protein structure and associated functional properties. Several cell culture experiments suggest that LAMP mediates axon targeting and growth in the brain (Keller et al., 1989; Pimenta et al., 1995; Mann et al., 1998; Gil et al., 2002).

## FUNCTIONAL STUDIES DEMONSTRATING A ROLE OF THE Lsamp GENE IN THE REGULATION OF EMOTIONAL BEHAVIOR

The first evidence for a role of the Lsamp gene in the regulation of emotional behavior came from a study where male Wistar rats were selected according to their exploratory behavior in the elevated plus-maze model of anxiety. Animals with lower exploratory activity (increased anxiety) had elevated levels of the Lsamp transcript in the periaqueductal gray (Nelovkov et al., 2003). In the same rats, an increase of Lsamp gene expression was also noticed in the amygdala, but not in the frontal cortex (Nelovkov et al., 2006). Exposure of rats to cat odor, another model of anxiety in rodents, also increased the expression of Lsamp transcript in the amygdala (Kõks et al., 2004). These findings were extended by Alttoa et al. (2010) demonstrating that the transcript for Lsamp was more expressed in the raphe, hippocampus, and frontal cortex of rats displaying reduced exploratory activity in the motility box. Lamprecht et al. (2009) established that fear conditioning caused changes in the Lsamp transcript expression in the amygdala of rats. Altogether, rodent studies indicate that increased level of the Lsamp transcript in several brain areas is related with increased trait anxiety (Nelovkov et al., 2003,^2006^; Alttoa et al., 2010), acute fear reaction (Kõks et al., 2004), and fear conditioning (Lamprecht et al., 2009).

## EVIDENCE FROM STUDIES WITH Lsamp-DEFICIENT MICE SUPPORTING ITS ROLE IN THE REGULATION OF EMOTIONAL BEHAVIOR

Two different Lsamp gene knockout mouse lines have been created. The first (Catania et al., 2008) was created by deleting exon 2 and the mice were back-crossed more than 10 generations onto the C57BL/6J background. The second (Innos et al., 2011) was created by our research team by deleting exon 1b and we used F2 hybrids [(129S6/SvEvTac × C57BL/6) × (129S6/SvEvTac × C57BL/6)] for the behavioral experiments, because the congenic footprint effect does not allow to achieve a pure background by backcrossing and besides, it has been argued that a robust behavioral phenotype, detectable over and above the behavioral variability caused by a mixed genetic background, obviates the need for back-crosses (Schalkwyk et al., 2007). Several cell culture experiments suggest that LAMP mediates axon targeting and growth in the brain (Keller et al., 1989; Mann et al., 1998; Gil et al., 2002). However, obvious lack of deviations in the brain organization of Lsamp-deficient mouse line, lacking exon 2 of the Lsamp gene, revealed that LAMP is rather mediating finely specialized aspects of circuit formation and maturation of the limbic system (Catania et al., 2008). These two mouse lines have remarkably similar phenotypes: both display no changes in sensory and motor development, are slightly hyperactive in novel environments, and perform more open arm entries and head-dips, and spend more time on open arms in the elevated plus-maze. However, there are also differences between the phenotypes: the first Lsamp gene knockout mouse line, generated in the Vanderbilt University (U.S.A.), exhibited a pronounced deficit in spatial memory acquisition in the water maze and poorly sustained CA1 long-term potentiation (Qiu et al., 2010). The second Lsamp gene knockout mouse line, generated by us, performed normally in the water maze and also displayed no learning deficiencies in the active avoidance test, another learning paradigm (Innos et al., 2011). We have also seen several unique phenotypic differences in our Lsamp gene knockout mice, such as decreased body weight, lack of whisker trimming, decreased aggressive behavior and reduced swimming speed (Innos et al., 2011). A possible explanation for the reduced agonistic and aggressive behavior could be the increased tone of 5-hydroxytryptamine (5-HT) system in the forebrain structures established in male Lsamp-deficient mice (Innos et al., 2013).

In the pharmacological studies we have found that the anxiolytic effect of diazepam is stronger in Lsamp-deficient mice compared to their wild-type littermates. Moreover, the ratio of GABA_A_ receptor subunit genes is shifted in favor of the alpha2 subunit compared to the alpha1 subunit in the temporal lobe of Lsamp-deficient mice. This could be a possible reason for the reduced anxiety as well as the increased anxiolytic effect of diazepam in Lsamp-deficient mice (Innos et al., 2011). Besides that the motor stimulant effect of amphetamine was significantly weaker in young Lsamp-deficient mice (Innos et al., 2013). Moreover, amphetamine did not cause place preference in these mice. The reduced action of amphetamine was accompanied by reduced expression of dopamine transporter, a major target of amphetamine, gene in the mesencephalon. The reduced behavioral effects were accompanied by increased levels and reduced turnover of 5-HT in the forebrain structures in response to amphetamine administration (Innos et al., 2013). Altogether, our recent pharmacological and biochemical studies point toward changes in the function of GABA-, 5-HT-, and dopaminergic systems in Lsamp-deficient mice.

## INFLUENCE OF ENVIRONMENTAL MANIPULATIONS ON THE BEHAVIOR OF Lsamp-DEFICIENT MICE

In order to study the impact of environmental manipulations on the phenotype, we exposed male Lsamp-deficient mice to environmental enrichment (EE), a technique that has often been shown to abolish phenotypic deviations in knockout mice, and to social isolation, a stressful manipulation, after which all the mice were tested in a behavioral battery. EE abolished differences between the genotypes in body weight and anogenital sniffing, a behavior related to aggressiveness, and amplified the anxiolytic-like phenotype of Lsamp-deficient mice both in the plus-maze and motility box (Innos et al., 2012). Isolation abolished differences between the genotypes in body weight and anxiety and amplified the differences in swimming speed and anogenital sniffing. EE and isolation failed to modify the results as compared to standard housing in whisker trimming, locomotor activity, marble burying, and corticosterone levels. In conclusion, Lsamp-deficient mice were less sensitive to isolation stress than their wild-type littermates. Lack of LAMP protein seemingly leads to a deterioration in the ability to adapt to novel stressful environments and stimuli (Innos et al., 2012).

## GENE POLYMORPHISM STUDIES DEMONSTRATING A ROLE OF LSAMP IN MOOD DISORDERS

Human data link LSAMP not only with anxiety, but also with a wider spectrum of psychiatric disorders: polymorphisms in the human LSAMP gene have been associated with panic disorder (PD; Koido et al., 2006) and male completed suicide (Must et al., 2008). Furthermore, the levels of LAMP protein have been found to be approximately 20% increased in postmortem frontal cortex both in patients with schizophrenia and bipolar disorder (Behan et al., 2009).

Our recent studies indicate that the LSAMP gene is possibly related to major depressive disorder (MDD) and PD (Koido et al., 2012). We detected statistically significant allelic associations between four LSAMP SNPs and MDD. Two SNPs out of these four contributed to the increased risk (rs1461131 and rs4831089) and two were protective (rs16824691 and rs9874470). Interestingly, all associated SNPs in our study reside in the first intron (1b) and may affect the regulation of gene expression. Only two risk SNPs above were related to PD. Haplotype block was formed from the forenamed SNPs giving three statistically significant haplotypes in the MDD group: TATA was a risk haplotype, whereas CGAG and CGTG were protective haplotypes. The risk haplotype TATA was also statistically significant in the PD group (Koido et al., 2012). On a closer analysis of frequencies of minor alleles of haplotype block, the risk haplotype TATA appeared more frequently than the protective haplotypes CGAG and CGTG in the MDD group (41 vs 24%) as compared to their respective control group (31 vs 33%; Pearson’s Chi square 10.96, *P* = 0.0009; Koido et al., 2012). The same trend was observed in the PD group where the risk haplotype TATA was more frequent than the protective haplotypes CGAG and CGTG (38 vs 28%) as compared to their respective control group (31 vs 34%). However, the difference in the case of PD did not reach to the level of statistical significance (Pearson’s Chi square 3.37, *P* = 0.066). Therefore, this study (Koido et al., 2012) underlines a significantly stronger relation of the LSAMP gene polymorphisms to MDD than to PD.

## STATE OF ART

In conclusion, the studies described above, performed in laboratory animals and psychiatric patients, suggest a possible role of LAMP in the mechanisms of psychiatric disorders. The Lsamp gene seems to be involved in the regulation of emotional behavior in rodents as the elevated function of the Lsamp gene is accompanied with increased anxiety, whereas the genetic invalidation of the Lsamp gene leads to the reduction of anxiety in rodents. There is also a shift in the expression of GABA_A_ receptor subunits in favor of alpha2. This could be a reason for the reduced anxiety and increased anxiolytic action of diazepam in Lsamp-deficient mice (Innos et al., 2011). Furthermore, the adaptation of Lsamp-deficient mice is impaired in novel and stressful environments and they display reduced aggressiveness. Also, their response to the behavioral effects of amphetamine is blunted (Innos et al., 2013). These behavioral effects are most likely related to changes in the function of the 5-HT-ergic system in Lsamp-deficient mice. Besides that we found a relation between gene polymorphisms of the LSAMP gene and MDD (Koido et al., 2012). Patients suffering from MDD have significantly increased ratio between the risk and protective haplotypes of the LSAMP gene compared to healthy subjects. The impact of these haplotypes for the function of LAMP is not clear and remains to be elucidated in future studies.

## Conflict of Interest Statement

The authors declare that the research was conducted in the absence of any commercial or financial relationships that could be construed as a potential conflict of interest.
